# MRI nomenclature for musculoskeletal infection

**DOI:** 10.1007/s00256-021-03807-7

**Published:** 2021-06-18

**Authors:** Erin F. Alaia, Avneesh Chhabra, Claus S. Simpfendorfer, Micah Cohen, Douglas N. Mintz, Josephina A. Vossen, Adam C. Zoga, Jan Fritz, Charles E. Spritzer, David G. Armstrong, William B. Morrison

**Affiliations:** 1 NYU Langone Medical Center, New York, NY USA; 2 UT Southwestern Medical Center, Dallas, TX USA; 3 Cleveland Clinic, Cleveland, OH USA; 4 Albert Einstein Medical Center, Philadelphia, PA USA; 5 HSS, New York, NY USA; 6 VCU, Richmond, VA USA; 7 Thomas Jefferson University Hospital, Philadelphia, PA USA; 8 Duke University Hospital, Durham, NC USA; 9 Keck Hospital of USC, Los Angeles, CA USA

**Keywords:** MRI, Musculoskeletal infection, Osteomyelitis, Abscess

## Abstract

The Society of Skeletal Radiology (SSR) Practice Guidelines and Technical Standards Committee identified musculoskeletal infection as a White Paper topic, and selected a Committee, tasked with developing a consensus on nomenclature for MRI of musculoskeletal infection outside the spine. The objective of the White Paper was to critically assess the literature and propose standardized terminology for imaging findings of infection on MRI, in order to improve both communication with clinical colleagues and patient care.

A definition was proposed for each term; debate followed, and the committee reached consensus. Potential controversies were raised, with formulated recommendations. The committee arrived at consensus definitions for cellulitis, soft tissue abscess, and necrotizing infection, while discouraging the nonspecific term phlegmon. For bone infection, the term osteitis is not useful; the panel recommends using terms that describe the likelihood of osteomyelitis in cases where definitive signal changes are lacking. The work was presented virtually to SSR members, who had the opportunity for review and modification prior to submission for publication.

## Introduction

Vocabulary describing musculoskeletal infection originally devised for radiographs has been adopted to magnetic resonance imaging (MRI), often without evidence or expert consensus. Thus, radiologists often use poorly defined and variably applied terms (i.e., osteitis) to describe infection, which may confuse referring physicians and often lack the scientific weight needed to guide medical and surgical decisions. Duryea et al. [1] found that MRI reports for pedal infection had little influence on clinical management.

The issue is magnified by problems associated with tissue sampling for definitive diagnosis of osteomyelitis, with culture yields as low as 21–28% [2, 3]. A pathologic diagnosis of osteomyelitis requires isolation of bacteria from a reliably obtained (uncontaminated) sample, along with histologic evidence of inflammatory cells and osteonecrosis [4]. However, insufficient sample, prior antibiotic therapy, or inability to culture an organism contribute to false-negative diagnoses, while false-positive diagnoses may arise from contaminants colonizing the skin or wound [4]. Meanwhile, many radiologists are justifiably reticent to biopsy bone if the needle has to traverse an area of cellulitis. Although we are unaware of any documented cases in the literature, it is possible that breeching the cortex in an area of soft tissue infection could actually result in an iatrogenic bone infection; so, certainty and clarity on MRI are paramount.

In the soft tissues, issues arise when intravenous contrast is not administered, causing difficulty in the differentiation between bland (noninflammatory) edema and cellulitis, and in the identification of soft tissue abscesses. A soft tissue abscess is often described as “drainable” based on imaging features, although this term is nebulous, and size, location, and morphologic criteria do not necessarily dictate whether an abscess is suitable for drainage. Issues in terminology arise from the nonspecific term phlegmon, which has been applied to both infectious and inflammatory conditions. Finally, difficulty arises in the overlapping imaging appearance of infectious and inflammatory arthritis, tenosynovitis, and bursitis.

Our goal was to use a panel of Musculoskeletal Radiologists to search the MRI literature for terms relevant to description of infection and clearly define these terms using evidence-based analysis and expert consensus. Recommendations of the panel will form a base for future research as well as clinical work, facilitating effective medical and surgical management decisions.

## Methods

The Society of Skeletal Radiology (SSR) Practice Guidelines and Technical Standards Committee identified musculoskeletal infection as a topic for study and selected an ad hoc White Paper Committee; twelve SSR member musculoskeletal radiologists and one podiatric surgeon with expertise in diabetic foot infection, tasked with developing a consensus on nomenclature for musculoskeletal infection. Musculoskeletal radiologists on the panel were selected by the SSR Practice Guidelines and Technical Standards Committee based on prior musculoskeletal infection research or clinical experience and expertise in MRI for musculoskeletal infection. A musculoskeletal radiologist with longstanding expertise and an author of many of the original publications on MRI of musculoskeletal infection was selected as the senior author and committee chair. We limited our study to MR imaging, and limited scope to infection outside of the spine (but including the sacroiliac joints). A literature search and conference call determined the range of terms used; the committee was divided into six subgroups. Subgroup categories and assigned terms are outlined in Table 1.

**Table 1:** Summary of terms assigned to each category

Category	Assigned terms
Soft tissue 1	Edema, cellulitis, ulcer, cloaca, sinus tract
Soft tissue 2	Soft tissue abscess, phlegmon, devitalized tissue, necrotizing fasciitis
Joints/tendon sheaths	Septic arthritis, synovitis, septic/infectious tenosynovitis, and erosion
Bone surface	Periostitis, periosteal reaction, periosteal new bone formation, subperiosteal abscess, cortical breakthrough
Medullary space	Osteomyelitis, osteitis, intra-osseous abscess
Necrosis	Sequestrum, involucrum

Each subgroup performed a literature review using the following inclusion criteria: original English language scientific papers pertaining to the terms assigned; preference for manuscripts with a study population > 10 patients. Group conference calls were utilized to discuss and debate terms and controversies and to formulate imaging recommendations. The committee reached casual consensus through group discussion, which we acknowledge may have potentially over-represented the recommendations of more vocal group members.

Following committee discussions, each subgroup contributed to the preliminary manuscript. The manuscript was subsequently organized to best present the definition and diagnosis of related terms, followed by potential controversies and rationale, and finally formulated recommendations, following a structure previously used for the SSR White Paper (Table 2). The work was presented virtually to SSR members for additional recommendations. After modification and society consensus, the final manuscript was submitted for publication.

**Table 2:** Summary of terms, controversy, and recommendations for musculoskeletal infection on MRI

Current term	Definition	Controversy and rationale	Recommendations
**Soft tissue**			
Edema	A local or generalized condition in which body tissues contain an excessive amount of fluid in the interstitial spaces Bland edema: non-inflammatory edema	Differentiation of bland edema from cellulitis	- Without intravenous contrast, confluent subcutaneous edema can be reported as **high or low likelihood of cellulitis** based on the presence or absence of secondary imaging features (i.e., ulcer, sinus tract, demarcated fluid collection) **Recommended term: edema**
Cellulitis	Non-necrotizing superficial bacterial infection	Differentiation of bland edema from cellulitis	- Cellulitis should be used for enhancing superficial soft tissues **Recommended term: cellulitis**
Ulcer	Breach in the continuity of skin, epithelium, or mucous membrane	Granulated ulcers may not have an identifiable skin breach but carry a similar risk of deep infection	**-** Accurately describe any associated soft tissue and/or osseous infection whenever an ulcer is visible on imaging or identified clinically **- Tailor field of view to region of concern** and **place markers adjacent to shallow ulcers** **Recommended term: ulcer**
Sinus tract	An abnormal channel that originates from the skin or a mucous surface to a deep-seated focus of suppuration	Squamous cell carcinoma is an uncommon complication that may develop in the sinus tract patient with longstanding osteomyelitis	**- Should be evaluated in all imaging planes** -While sinus tract is not specific to infection, it is an appropriate term for processes isolated to the soft tissues **Recommended term: sinus tract**
Soft tissue abscess	Localized collection of pus in any body part resulting from invasion of a pyogenic bacterium or other pathogen, with a peripheral capsule created by macrophages, fibrin, and granulation tissue	Difficult to discern without intravenous contrast or diffusion-weighted imaging (DWI)	- Soft tissue abscess should be used for demarcated fluid collections with peripheral enhancement or with restricted diffusion or presence of penumbra sign if contrast is not administered **- Intramuscular abscess** is preferred term if there is a defined walled-off intramuscular fluid collection - Avoid classifying an abscess as drainable based on imaging features - Histopathology terms, such as tissue necrosis, liquefied necrosis or tissue infarction are discouraged **Recommended terms: soft tissue abscess, intramuscular abscess**
Phlegmon	Acute or infiltrative phase ill-defined inflammatory mass-like lesion, prior to liquefaction and pseudocapsule formation (pre-abscess)	- Does not specify the presence of infection - DWI may show abscess in a region of phlegmonous change	- The term phlegmon is discouraged as it would not lead to meaningful clinical action or impact **Recommended terms: rather than phlegmon, use accepted terms such as cellulitis, myositis, or fasciitis, without soft tissue abscess**
Devitalized tissue	Necrotic or ischemic soft tissue	- Only visible after contrast administration - Unclear whether tissue is truly necrotic or ischemic	**-Recommend contrast administration** to reveal devitalized tissue and optimize resection - Carefully scrutinize the soft tissues underneath or beyond ulcer margin for devitalized tissue **Recommended term: devitalized tissue**
Necrotizing fasciitis	An aggressive bacterial infection involving subcutaneous fat and deep fascial compartments	Clinical and imaging findings overlap with non-necrotizing fasciitis, pyomyositis, cellulitis with vascular thrombosis, prior radiation treatment	- **CT should be recommended as the initial imaging study. The presence of gas bubbles is diagnostic, while their absence does not exclude this diagnosis** **Recommended term: necrotizing deep soft tissue infection**
**Joints/tendon sheaths**			
Septic arthritis	Intra-articular infection	Imaging appearance similar to inflammatory or crystal arthropathies	- **Any monoarticular destructive arthropathy should be regarded as septic arthritis until excluded** **-** The term infectious or inflammatory arthritis may be used if the diagnosis is uncertain - Specific terms for type of infection of specific joint should be avoided - Acceptable to use term **septic sacroiliitis** **Recommended terms: septic arthritis, septic sacroiliitis, or infectious or inflammatory arthritis when the diagnosis is uncertain**
Synovitis	Inflammation of the synovial-lined spaces of joints, bursae, or tendon sheaths	Nonspecific term applying to infectious and non-infectious conditions	**- When reported, recommend including a differential diagnosis including an estimation of risk** **Recommended term: synovitis**
Septic or infectious tenosynovitis	Infection of tendon sheath	Imaging overlap with tenosynovitis due to inflammatory or crystal arthropathies	- Septic or infectious tenosynovitis may be used when imaging findings match clinical picture - Term should be avoided in tendons without sheath (i.e., Achilles). Instead, infectious paratenonitis or infection of a specific tendon (i.e., infection of the Achilles tendon) recommended **Recommended terms: Septic or infectious tenosynovitis, infectious paratenonitis, or infection of tendon (in tendons without a sheath)**
Erosion	Loss of subchondral bone plate integrity	- May represent early stage of medullary involvement and osteomyelitis in the context of septic arthritis - Also seen in inflammatory or crystal arthropathies	- May be used in the context of septic arthritis, with caveat of possible early osteomyelitis, particularly with extension beyond the immediate subchondral bone **Recommended term: erosion**
**Bone surface**			
Periosteal reaction, periostitis, periosteal new bone formation	The reaction of periosteum to abnormal stimulants by forming new bone in distinctive patterns	Paucity of MRI literature on term due to lower MR spatial resolution, marrow signal changes specific for osteomyelitis	**Recommended term: periosteal reaction**
Subperiosteal abscess	Encapsulated fluid collection confined to the subperiosteal space	May be difficult to differentiate subperiosteal abscess from phlegmon	**Recommended term: subperiosteal spread of infection**
Cloaca	Opening or rupture of bone cortex overlying an area of osteomyelitis, allowing discharge of granulation tissue, pus, or necrotic bone	- Remnant of reparative callus may persist within the cortex after the infection has cleared - Should be differentiated from pathologic fracture and erosion	- Cloaca should be used for an opening or rupture of cortex overlying an area of osteomyelitis - Pathologic fracture should be used when there is a delineated fracture cleft resulting from weakened bone undergoing minimal stress - The nonspecific term cortical breakthrough is discouraged but may be used when the etiology is unclear - **Consider repeat MRI** to identify intraosseous fluid collections deep to the cloaca as an indicator of persistent infection **Recommended term: cloaca**
**Medullary space**			
Osteomyelitis	Infection of bone which involves the medullary canal	- Discordant marrow signal - Concomitant trauma, neoplasia, arthropathies, or osteonecrosis - Marrow signal abnormality in neuropathic arthritis difficult to differentiate from osteomyelitis - Vascular insufficiency may fail to produce T1 marrow replacement, enhancement	- **Osteomyelitis** is recommended when concordant marrow signal changes are present - “Osteitis” and “reactive marrow edema” should be avoided in infection, but still apply in non-infection cases like those due to inflammatory arthritis - “**High likelihood of osteomyelitis**" is recommended for any hyperintense marrow signal on fluid-sensitive images(regardless of T1 signal) adjacent to an ulcer, abscess, or sinus tract - **Chronic osteomyelitis** should be used if the marrow demonstrates patchy areas of active disease and fibrosis, especially with features such as cortical remodeling, Brodie’s abscess, sequestrum, or sinus tract - **Infected, devitalized bone** may be appropriate when there are findings diagnostic of osteomyelitis without contrast enhancement **Recommended terms: osteomyelitis, “high or low likelihood of osteomyelitis,” chronic osteomyelitis, infected-nonviable bone**
Intraosseous abscess	Intraosseous cavity filled with pus, with rim of granulation tissue	- May be difficult to differentiate between intraosseous abscess and neoplasia	- **Intraosseous abscess** is appropriate for intraosseous fluid-signal cavities with a rim of peripheral enhancement, or in the presence of restricted diffusion or the penumbra sign if contrast not administered - **Brodie’s abscess** should be used for intraosseous abscesses in subacute or chronic osteomyelitis having a predilection for the ends of tubular bones **Recommended terms: intraosseous abscess, Brodie’s abscess**
**Necrosis**			
Sequestrum	Devitalized bone sequestered from viable bone in chronic osteomyelitis	Presence of a sequestrum is not definitive for infection	- Sequestrum should be used for an area of necrotic bone surrounded by viable, infected bone, often having a rim of granulation tissue **Recommended term: sequestrum**
Involucrum	Formation of a capsule of viable, new bone around an area of sequestered, necrotic bone		- Involucrum should be used to describe a capsule of viable, new bone which forms around an area of necrotic (sequestered) bone **Recommended term: involucrum**

### Intravenous contrast in renal failure

Intravenous contrast is an important diagnostic tool in MRI for musculoskeletal infection. An association has been noted between gadolinium-based contrast agents and the development of nephrogenic systemic fibrosis in patients with end-stage chronic kidney disease, particularly in patients on dialysis. Given that many patients with musculoskeletal infection have diabetes or renal insufficiency, it is important to carefully consider risk of nephrogenic systemic fibrosis in this cohort [5].

Gadolinium-based contrast agents differ in the propensity for gadolinium to dissociate from chelates in at-risk patients, leading to a varying risk profile for the development of nephrogenic systemic fibrosis. Based on the available evidence, group 2 agents (MultiHance-Bracco Diagnostics, Gadavist-Bayer HealthCare Pharmaceuticals, Dotarem-Guerbet, Clariscan-GE Healthcare, ProHance-Bracco Diagnostics) are associated with the lowest risk, such that assessment of renal function with a questionnaire or laboratory testing is optional prior to administration. Patients receiving group 1 agents (Omniscan-GE Healthcare, Magnevist-Bayer HealthCare Pharmaceuticals, OptiMARK-Guerbet) should be considered at risk for developing nephrogenic systemic fibrosis if they are on dialysis, have severe or end-stage renal failure (eGFR < 30 mL/min/1.73 m^2^) without dialysis, or have acute renal injury. There is insufficient evidence on the administration of group 3 agents (Eovist-Bayer HealthCare Pharmaceuticals), and thus risk of developing nephrogenic systemic fibrosis should be considered in patients with risk factors delineated above [5].

In patients unable to receive intravenous contrast, ancillary diagnostic tools or alternative modalities may be considered, including MRI with diffusion-weighted imaging (DWI), ultrasound, or CT.

## Soft tissue

### Edema and cellulitis

#### Definition and diagnosis

Edema is defined as a local or generalized condition in which body tissues contain an excessive amount of tissue fluid in the interstitial spaces [6]. Bland edema is defined as noninflammatory edema. Noninflammatory causes of soft-tissue edema include congestive heart failure, diabetic vascular insufficiency, lymphatic obstruction, and venous thrombosis. Inflammatory causes of soft tissue edema include trauma, hypersensitivity response, and infection [7].

Cellulitis is a non-necrotizing bacterial infection limited to the superficial soft tissues (the skin, subcutaneous fat, and superficial fascia (fascial layer deep to the skin and subcutaneous fat)), without deep soft tissue (muscular or deep fascial (fascial layer enveloping muscle)) involvement, while necrotizing fasciitis demonstrates deep fascial and muscle involvement [7, 8]. Cellulitis is most often caused by β-hemolytic streptococci, followed by methicillin-sensitive *Staphylococcus aureus* or methicillin-resistant *S. aureus*, particularly in high-risk populations [9]. Cellulitis presents with local erythema, warmth, swelling, and tenderness, with systemic signs of fever and leukocytosis, and occurs most commonly in the lower legs (below the knee) [10]. Bacteria can be introduced through an area of open skin, but in some cases, there is no obvious entry site.

**Fig. 1:**
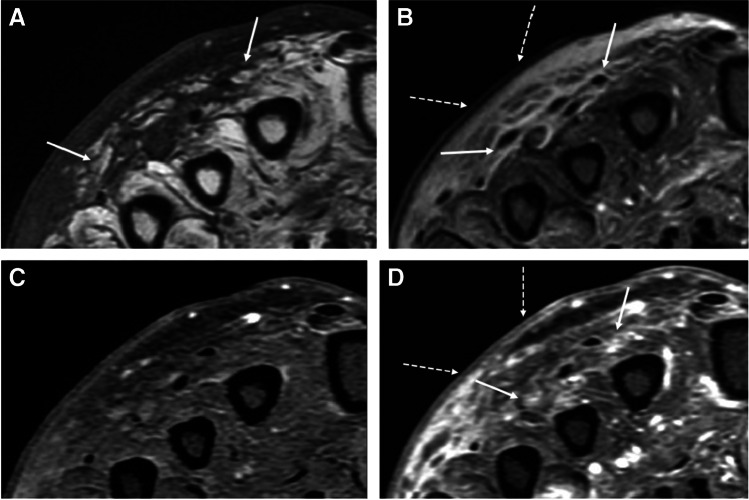
Cellulitis of the foot in a 61-year-old male. Short axis T1 (**A**) and proton-density fat-suppressed images (**B**) show skin thickening (dashed arrows, **B**) and cellulitis of the superficial subcutaneous tissues, with edema-like signal and reticulation of the subcutaneous fat (arrows **A**, **B**). Pre-contrast (**C**) and post-contrast (**D**) fat-suppressed T1 images show ill-defined enhancement of the skin (dashed arrows, **D**) and superficial subcutaneous tissues (arrows, **D**)

MRI is the most accurate and specific imaging modality for confirming the presence and extent of cellulitis [11–13]. Edema and cellulitis present as reticulated T1 hypointense, fluid-sensitive sequence hyperintense signal in the subcutaneous fat and superficial fascia [14, 15], in a confluent (diffuse) or focal pattern, with only cellulitis demonstrating ill-defined diffuse enhancement, along with thickening of the skin and enlargement of septa in the subcutaneous fat (Fig. 1) [16–19]. The extent of enhancement in cellulitis depends to some degree on the delay in image acquisition.

#### Controversy and rationale

**Fig. 2:**
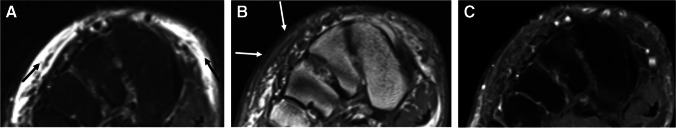
Bland edema in a 59-year-old male. Short axis T2 fat-suppressed (**A**), T1 (**B**), and T1 post-contrast images with fat-suppression show confluent subcutaneous edema at the dorsum of the foot (arrows, **A**), with thickening of the dermis (arrows, **B**) but no visible skin defect or organized fluid collection. Lack of enhancement on post-contrast image (**C**) confirms the diagnosis of bland edema

Bland edema is occasionally difficult to differentiate from cellulitis when signal abnormality is noted within the superficial soft tissues without morphologic abnormality (i.e., ulcer, sinus tract, fluid collection) in patients for whom intravenous contrast was not administered or contraindicated (Fig. 2). In this setting, ancillary findings may be useful to support a diagnosis of either bland edema (i.e., bilateral or circumferential edema, recent trauma, history of congestive heart failure, low albumin, or nephrotic syndrome) or cellulitis (i.e., focal, unilateral edema, elevated white blood cell count, elevated inflammatory markers, fever).

Intravenous contrast is recommended, if not contraindicated, to differentiate abscess from focal cellulitis and other noninfectious causes of subcutaneous edema, with the caveat that tissue enhancement will be potentially delayed in patients with vascular insufficiency. In this patient population, consider adding delayed post-contrast imaging [20]. If intravenous contrast is contraindicated, DWI may be a useful adjunct diagnostic tool, particularly in the diagnosis of soft tissue abscesses, which will show restricted diffusion.

#### Recommendations

- The term cellulitis can be used when there is enhancement of the superficial soft tissues following intravenous contrast administration.
- Without intravenous contrast, confluent subcutaneous edema can be reported as high or low likelihood of cellulitis based on the presence or absence of ancillary findings and secondary imaging features of infection (i.e., ulcer, sinus tract, demarcated fluid collection).

### Ulcer

#### Definition and diagnosis

Ulcer is a breach in the continuity of skin, epithelium, or mucous membrane, that may be limited to the epidermis, or may extend from the epidermis with deeper involvement of the dermis, subcutaneous fat, or the deep soft tissues. Diabetic patients are particularly susceptible to developing foot ulcers from cumulative mechanical trauma, and immobile patients are prone to pressure ulcers about the pelvis, heels, and other high-risk areas. Skin ulcers compromise the natural defense of the integumentary system and lead to local eschar and scar formation as well as poor perfusion, creating an ideal substrate for bacterial reproduction and invasion, which in severe cases may lead to amputations or systemic infection. Severe wound infection is characterized by local infection with signs of systemic inflammatory response syndrome. Presence of ischemic tissue increases the severity of infection, with critical ischemia often indicative of severe infection [4]. Gram-positive bacteria are the most common isolated pathogens in most Western nations, although severe wounds have a greater likelihood of Gram-negative or anaerobic bacteria [4, 21, 22].

Wounds with significant nonviable tissue or extensive osseous or articular involvement are usually indicated for surgical intervention. A more urgent surgical intervention is usually recommended for wounds with deep tissue gas, abscess formation, or necrotizing fasciitis. All diabetic patients with foot wounds should receive targeted wound care including debridement, pressure offloading, and adequate wound dressing [4].

MRI can optimize planning for patients indicated for surgery and can minimize the area of resection by mapping extent of the ulcer and soft tissue infection [23, 24]. A marker should be placed adjacent to shallow ulcers that may not be visible at imaging. The field of view should be tailored to include the entirety of the ulcer and soft tissue infection.

**Fig. 3:**
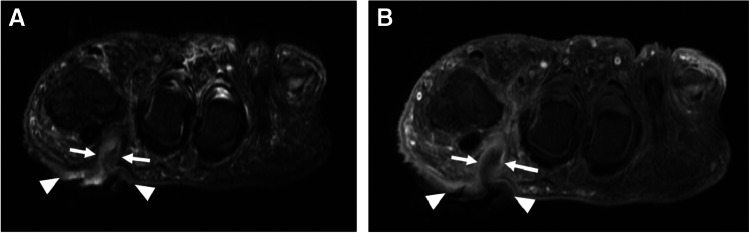
Plantar ulcer and sinus tract in a 55-year-old male. Short axis T2 fat-suppressed (**A**) and T1 fat-suppressed post-contrast images (**B**) demonstrate ulceration of the plantar soft tissues underlying the first webspace (arrowheads **A**, **B**), with contiguous sinus tract (arrows **A**, **B**) outlined by thin enhancing granulation tissue

MRI imaging manifestations include focal skin disruption with elevated margins, with an associated soft-tissue defect, demonstrating hyperintense signal on fluid-sensitive images, with marked peripheral enhancement, a finding indicative of granulation tissue at the base of the ulcer (Fig. 3). Nonenhancing tissue overlying an area of ulceration should be recognized and reported as nonviable eschar that can be targeted for debridement.

#### Controversy and rationale

MRI is not utilized for the diagnosis of soft tissue ulcer, which is apparent on physical exam alone. Rather, MRI is ordered in patients with soft tissue ulcer to evaluate the extent of soft tissue infection, and any osseous or articular involvement.

Granulated ulcers without visible soft-tissue defects should be carefully reviewed, as the likelihood of a deep infection is similar to that of an open ulcer [25]. Both sterile granulation tissue and soft tissue infection show hyperintense signal on fluid-sensitive images and enhance with intravenous contrast [26]. In this setting, relative indicators of infection include direct continuity of the tissue with a skin ulcer, soft tissue gas, and contained fluid collections.

#### Recommendations

- Markers should be placed adjacent to the ulcer, with field of view centered on area of concern.
- Accurately describe any associated soft tissue and/or osseous infection whenever an ulcer is visible on imaging or identified clinically.

### Sinus tract

#### Definition and diagnosis

A sinus tract is an abnormal channel that originates from the skin or a mucous surface to a deep-seated focus of suppuration [27]. A soft tissue ulcer can show a sinus tract leading to a deeper soft tissue abscess and may occasionally extend to a joint. Chronic osteomyelitis can be associated with a sinus tract and cloaca (opening or rupture of bony cortex overlying an area of osteomyelitis) draining granulation tissue, pus, or necrotic debris from the bone to the skin.

MRI findings of sinus tract include a linear structure which may contain fluid, granulation tissue, or necrotic debris extending from bone or soft tissues to the skin surface, with T1 hypointense signal, hyperintense (fluid) signal on fluid-sensitive images, and a “tram-track” pattern of peripheral enhancement on post-contrast images (Fig. 3) [28, 29].

#### Controversy and rationale

The term sinus tract is not specific to infection, but in the setting of soft tissue or osseous infection, an identified sinus tract generally maps the extent of the infection. Squamous cell carcinoma is an uncommon complication that may develop in the sinus tract of patients with longstanding chronic osteomyelitis [30, 31].

#### Recommendations

- Sinus tracts should be evaluated in all imaging planes.
- While sinus tract is not specific to infection, it is an appropriate term for processes isolated to the soft tissues.

### Soft tissue abscess

#### Definition and diagnosis

A soft tissue abscess is a localized collection of pus in any body part resulting from invasion of a pyogenic bacterium or other pathogen, with a peripheral capsule created by macrophages, fibrin, and granulation tissue [6]. Common organisms include *Staphylococcus aureus*, streptococcus, *Serratia marcescens*, and *Pseudomonas aeruginosa*.

**Fig. 4:**
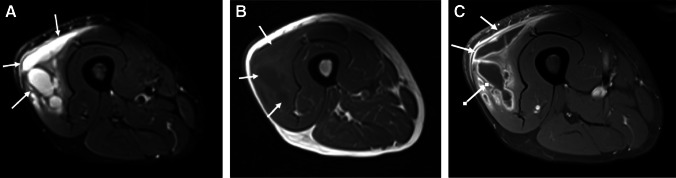
Thigh intramuscular soft tissue abscess in a 33-year-old male. Axial STIR (**A**), axial T1 (**B**), and axial T1 fat-suppressed post-contrast (**C**) images demonstrate an intramuscular multiloculated fluid collection within the lateral thigh (arrows, **A**), involving the vastus lateralis and rectus femoris muscles, demonstrating a subtle relatively T1 hyperintense rim (“penumbra sign,” arrows, **B**), and avid peripheral rim enhancement (arrows, **C**), compatible with an intramuscular soft tissue abscess

MR imaging is the preferred modality for soft tissue abscess [32, 33], with a reported sensitivity of 97%, and specificity of 77% [32]. On MRI, an abscess demonstrates a well-circumscribed area of T1W isointense or hypointense signal, hyperintense (fluid-like) signal on fluid-sensitive sequences, with T1W post-contrast rim enhancement (Fig. 4), with good to substantial inter-observer performance for detection [34]. The abscess cavity is surrounded by a fibrotic capsular rim which is low signal on pre-contrast MRI [33]. However, subacute, chronic, or acute on chronic abscesses may demonstrate a peripheral rim of relatively hyperintense signal relative to the low-signal abscess cavity on T1W pre-contrast images, referred to as the “penumbra sign,” with sensitivity and specificity of 54% and 98%, respectively, for differentiation of soft tissue infection from neoplasm [35]. Tissue displaying the penumbra sign seen at the periphery of abscess cavities represents highly vascularized granulation tissue, and demonstrates relatively high T1 signal due to the high protein content, with the membrane composed of leukocytes, lymphocytes, plasma cells, fibroblasts, and fibrillar connective tissue[36].

**Fig. 5:**
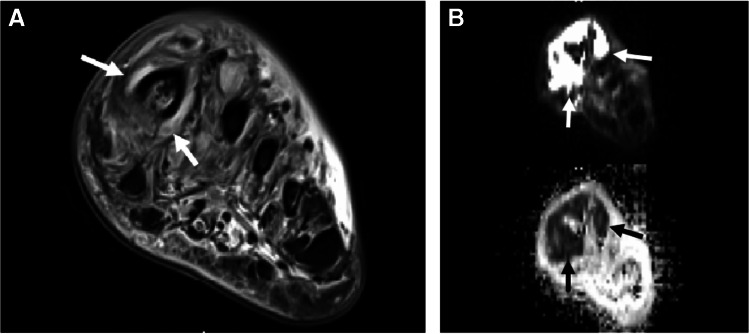
Utility of diffusion weighted imaging for abscess detection in a 47-year-old female. Short axis T2 Dixon water map image of the foot demonstrates a fluid collection encircling the first metatarsal (arrows, **A**), demonstrating high signal on diffusion-weighted images (arrows, **B**, image above, b = 800), and low signal (ADC = 0.5–0.6 × 10^−3^ mm^2^/s) on the ADC map (arrows, **B**, image below), features compatible with abscess

Abscesses are much more conspicuous on post contrast imaging, with increased reader confidence shown in both diagnosis and exclusion of the lesion in about 46–50% cases [17, 34, 37]. Intravenous contrast delineates necrotic nonenhancing abscess contents, which can be otherwise masked in the mound of hyperintense edema on the fluid-sensitive sequences [18, 19, 38]. Subtraction images may be obtained after contrast administration to increase conspicuity of a soft tissue abscess. For patients in whom contrast is contraindicated, adding DWI to conventional MRI enhances soft tissue abscess detection (Fig. 5) [39]. Most abscesses occur near a skin ulcer, or at the sites of osteomyelitis [20], and may be present in subcutaneous, fascial, or intramuscular tissue planes. Effective communication of the presence of a soft tissue abscess is paramount.

#### Controversy and rationale

Diagnostic difficulty on the MRI diagnosis of abscess arises when there is no intravenous contrast or DWI imaging, or when no rim enhancement is seen on contrast imaging. Contrast images may demonstrate both thick and thin rim enhancement, and it may not be possible to distinguish a sterile fluid collection or peripherally enhancing soft tissue mass (i.e., post-operative seroma, evolving hematoma, developing myositis ossificans, necrotic tumor, ganglion, foreign body reaction) from abscess [38, 40]. Gas can be seen more commonly in pyogenic abscesses. Diabetic versus non-diabetic abscesses, as well as tubercular and fungal abscesses show similar appearances and underlying clinical picture is paramount to arrive at such diagnoses [41, 42]. Other terms used in this domain include tissue necrosis or pyomyositis [43, 44]. Pyomyositis is a primary infection of the muscle but can also occur from a contiguous infection from the skin ulcer or sinus tract. The abscess characteristics on MRI are similar to what has been described above, with presence of focal hyperintense areas on fluid sensitive images showing rim enhancement and diffusion restriction, with underlying muscle changes of inflammation and edema.

The term “drainable soft tissue abscess” is nebulous and poorly defined. The Society of Interventional Radiology recommends image-guided percutaneous drainage or aspiration of abscesses and abnormal fluid collections for the following indications: suspicion that the fluid is infected or the result of abnormal fistulous communication, need for characterization, suspicion that the collection is producing symptoms sufficient to warrant drainage, or need for an adjunctive procedure to facilitate the improved outcome of a subsequent intervention [45]. Given importance of clinical context, and lack of specific size criteria, location, or morphologic imaging features identifying an abscess as drainable, we recommend avoiding usage of this term in MRI reports.

#### Recommendations

- The term soft tissue abscess should be used for demarcated fluid collections with peripheral enhancement, or with restricted diffusion or the presence of penumbra sign if contrast is not administered.
- Intramuscular abscess is a preferred term if there is a defined walled-off intramuscular fluid collection.
- Avoid characterizing an abscess as drainable or not drainable based on imaging features.
- Histopathology terms, such as tissue necrosis, liquefied necrosis, or tissue infarction are discouraged.

### Phlegmon

#### Definition and diagnosis

**Fig. 6:**
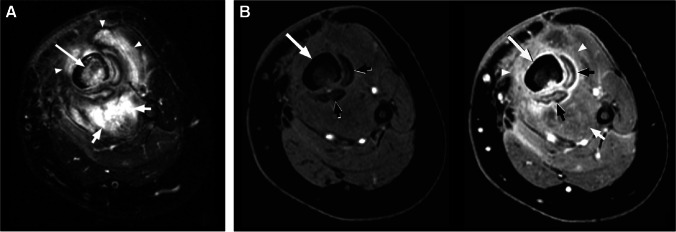
Involucrum and sequestrum in the lower leg of a 4-year-old male with chronic osteomyelitis. Axial T2-weighted fat-suppressed MR image (**A**) and corresponding axial pre and post-contrast T1-weighted fat suppressed MR images (**B**) of the lower leg show diffuse edema within the tibia (long arrow) with lack of enhancement, consistent with sequestrum formation. Surrounding muscular edema and enhancement (arrowheads, short white arrows) represents myositis, without discrete soft tissue abscess. The shell of enhancing bone (short black arrows) represents the new bone formation (involucrum)

Phlegmon (pre-abscess or immature abscess) is an ill-defined inflammatory mass-like lesion reflecting the acute or infiltrative phase of infected soft tissue, prior to liquefaction and pseudocapsule formation. Phlegmon appears as a non-encapsulated ill-defined area of low T1 and intermediate to high signal on fluid-sensitive images. Following intravenous contrast, there is variable enhancement without a discrete capsule or rim enhancement, which clinically implies that there is no abscess (Fig. 6) [46, 47].

#### Controversy and rationale

The term phlegmon gained acceptance in the setting of retroperitoneal inflammation in the setting of pancreatitis, mediastinal, and head and neck infections; however, this term is discouraged in those locations as well [48], since it does not specify the presence of infection or necrosis. DWI is a useful adjunct tool in delineating an abscess, which will demonstrate a discrete area of restriction on the ADC map, in a region of phlegmonous change.

#### Recommendations

- The term phlegmon is discouraged as it would not lead to meaningful clinical action or impact. We recommend using terms such as focal cellulitis, myositis (inflammation of muscle) [49], and fasciitis (inflammation of fascia) [6], without soft tissue abscess.

### Devitalized tissue

#### Definition and diagnosis

**Fig. 7:**
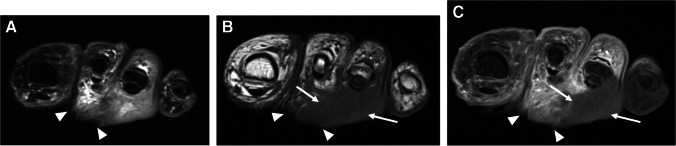
Devitalized tissue in an 83-year-old diabetic female. Short axis STIR (**A**), T1 (**B**), and T1 fat-suppressed post-contrast (**C**) images of the forefoot demonstrating shallow ulceration of the plantar soft tissues (arrowheads), with surrounding cellulitis, and a geographic area of non-enhancement (arrows **B**, **C**), compatible with devitalized tissue

Devitalized tissue occurs almost exclusively in the diabetic foot or in peripheral vascular disease. The authors prefer the umbrella term devitalized tissue to include necrotic or ischemic soft tissue. It can be reliably identified on contrast enhanced MRI as areas of non-enhancing tissue without rim enhancement [50]. The non-enhancing areas show homogeneous low signal after contrast, often with an abrupt cutoff of enhancement demarcating the viability border (Fig. 7). Subtraction images may be useful to make devitalized tissue more conspicuous on contrast-enhanced images. Devitalized tissue can be seen in up to one fourth of diabetic foot infections [20], and is essential to report, since complete resection of necrotic tissue promotes successful wound healing [51, 52].

#### Controversy and rationale

The term devitalized tissue has not been widely circulated in the literature. The reason might be that devitalized tissue is only visible after contrast administration, which may or may not be administered as part of the protocol, depending on the institution. In addition, it is not clear whether the non-enhancing component truly reflects necrosis, or simply ischemia due to arterial disease or venolymphatic congestion, and in this cohort, delayed contrast-enhanced images may be useful [20]. Also, it is possible that pressure on an extremity (i.e., related to MRI coil placement) may temporarily decrease perfusion and mimic necrosis. More studies with pathologic correlation are needed to determine the accuracy of prospective imaging in determining devitalized tissue short of histologic sampling.

#### Recommendations

- Intravenous contrast is recommended to reveal devitalized tissue and optimize resection.
- The term devitalized tissue should be used for geographic areas of soft tissue nonenhancement, especially underneath and beyond the ulcer margins.

### Necrotizing fasciitis

#### Definition and diagnosis

Necrotizing fasciitis is an aggressive bacterial infection involving subcutaneous fat and deep fascial compartments [49]. As opposed to uncomplicated infectious cellulitis or fasciitis, necrotizing fasciitis can be rapidly fatal if not promptly diagnosed or treated with surgical debridement [8]. The diagnosis is based on clinical and/or supportive imaging findings. Clinical presentation can be very similar to non-necrotizing fasciitis, cellulitis or myositis. Clinical features of necrotizing fasciitis include swelling, pain, and erythema, with more advanced cases demonstrating bullae, skin necrosis, and crepitus, with fever present in only 40% of cases [53]. The definite diagnostic criterion is surgical exploration depicting necrotic fat with brownish color and lack of resistance to manual debridement along the deep fascial plane. The LRINEC (laboratory risk indicator for necrotizing fasciitis) score is used clinically, consisting of a 13-point scoring system based on the results of routine laboratory tests (C-reactive protein, total white cell count, hemoglobin, sodium, creatinine, and glucose). Patients with a score of ≥ 6 should raise suspicion for necrotizing fasciitis, while a score of ≥ 8 is strongly predictive [54].

**Fig. 8:**
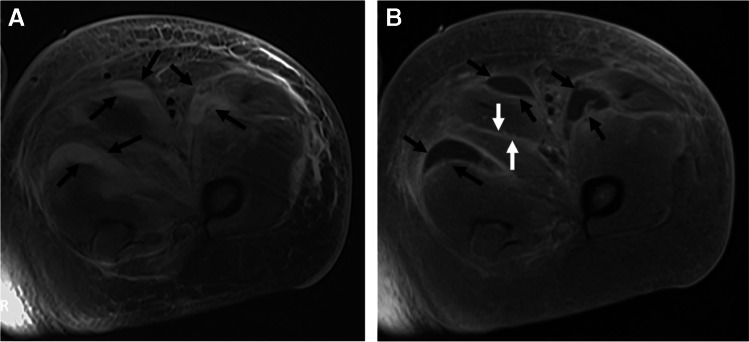
A 39-year-old female with necrotizing deep soft tissue infection of the thigh. Axial T2 fat-suppressed (**A**) and T1 fat-suppressed post-contrast (**B**) images of the thigh suggest presence of a necrotizing soft tissue infection, with rim-enhancing abscesses extending along deep fascial planes of multiple compartments (arrows **A**, **B**), with thick enhancement of the deep fascia (white arrows, **B**)

CT has the advantage of speed, availability, lack of required intravenous contrast, and ability to reliably identify even small amounts of soft tissue gas in necrotizing fasciitis compared to MRI. CT findings include fascial thickening, fat infiltration, focal fluid collections and soft tissue gas, although gas is seen in less than 50% of cases [55, 56]. On MRI, deep fascial thickening, fascial fluid pockets, heterogeneous fascial enhancement, fascial gas pockets, and peripheral band-like limited muscle edema and/or enhancement in a swollen extremity or trunk are suggestive of necrotizing fasciitis in the setting of increased serology markers of CRP, ESR, and white cell count (Fig. 8) [14, 15, 57, 58]. Kim et al. reported significantly greater frequency of findings, such as thickened (≥ 3 mm) deep fascia, deep fascial low signal on fat suppressed fluid-sensitive imaging with gas pockets, focal or diffuse non-enhancement of deep fascia, and involvement of ≥ 3 compartments in one extremity in necrotizing fasciitis as compared to non-necrotizing fasciitis [55]. On the contrary, in the absence of deep fascial abnormality, MRI excludes necrotizing fasciitis with excellent negative predictive value [15]. Yoon et al. integrated MRI findings with LRINEC score for differentiating necrotizing fasciitis from non-necrotizing fasciitis. The area under the (receiver operating characteristic) curve (AUC) was 0.814 (95% CI, 0.727–0.900; *p* < 0.001) for the LRINEC score alone, and 0.862 (95% CI, 0.787–0.938; *p* < 0.001) for the integrated model using two important MRI features—thickening of the deep fascia ≥ 3 mm and multi-compartmental involvement [59].

#### Controversy and rationale

MRI for evaluation of necrotizing soft tissue infection demonstrates high sensitivity, but low specificity [60]. Diagnostic dilemma arises as many clinically related and unrelated conditions, such as non-necrotizing fasciitis, pyomyositis, cellulitis with vascular thrombosis, and recent radiation treatment present similarly on MRI, and deep fascial thickening, fluid pockets, and enhancement may be observed in all such conditions, limiting the imaging evaluation [15, 61].

The disease process is also named differently depending upon the anatomic site resulting in confusion in terminology and clinical diagnosis, e.g., Fournier gangrene at the perineum, Ludwig angina at the submandibular region, gas-forming myonecrosis, etc. [60].

The LRINEC score in isolation exhibits moderate accuracy for necrotizing soft tissue infection. Although an integrated predictive model appears to be the most accurate [59], no single criteria can unequivocally diagnose or exclude, as identification of necrosis in the mound of inflammation and edema might be beyond the resolution of current imaging [57]. Lack of deep fascial involvement is useful in excluding necrotizing soft tissue infection. Given the low specificity of MRI, correlation with clinical findings (i.e., the LRINEC score) is paramount [15].

#### Recommendations

- Necrotizing deep soft tissue infection is the preferred more encompassing term, delineating involvement of both the deep fascia and musculature.
- CT should be recommended as the initial imaging study due to speed, ease of use, and lack of required intravenous contrast. The presence of gas bubbles is diagnostic, while their absence does not exclude this diagnosis.

## Joints/tendon sheaths

### Septic arthritis

#### Definition and diagnosis

Septic arthritis is a widely used term for intra-articular infection, and part of standard nomenclature. On MRI, septic arthritis is characterized by joint effusion (often complex, depending on chronicity) although this finding is obviously nonspecific. Septic arthritis may occur due to a hematogenous spread of infection, from contiguous spread, or from instrumentation (i.e., aspiration, arthrography, arthroscopy).

The most common organisms observed in septic arthritis are: *Staphylococcus aureus*, alpha- and beta-hemolytic streptococci, pneumococci, *Haemophilus*, *Pseudomonas*, gonococcus, *Escherichia coli*, and *Serratia* [62]. In the USA, septic arthritis from the tick-borne spirochete *Borrelia burgdorferi* (Lyme disease) is an important differential consideration [63]. In the periprosthetic joint, *Staphylococcus aureus*, coagulase-negative staphylococci, and streptococci are frequently observed, while *Propionibacterium acnes* may be observed in the periprosthetic shoulder [64].

From birth to approximately 1 year of age, metaphyseal and diaphyseal vascular channels traverse the physis and extend to the epiphysis, allowing for a focus of metaphyseal osteomyelitis to spread to involve the joint space. Vascular channels traversing the physis obliterate in children; however, slow metaphyseal flow predisposes children to osteomyelitis of the metaphysis, and transcortical extension may result in a septic joint if the physis is intraarticular. After physeal fusion in adults, nutrient vessels re-establish access to the epiphysis, allowing osteomyelitis to extend into the joint space [62]. Prosthetic joint infections and other peri-implant infections are important clinical entities in adults but are beyond the scope of this White Paper.

**Fig. 9:**
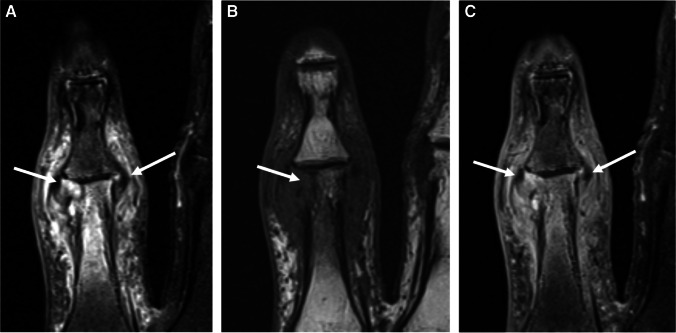
Septic arthritis and osteomyelitis in a 67-year-old female. Coronal T2 fat-suppressed (**A**), T1 (**B**), and T1 fat-suppressed post-contrast (**C**) images of the second digit show marked edema and enhancement of the proximal interphalangeal joint capsule and the surrounding soft tissues secondary to synovitis from septic arthritis (arrows, **A**, **C**), with symmetric, diffuse joint space narrowing and an erosion along the proximal phalanx head (arrow, **B**), with adjacent T1 marrow replacement and periosteal reaction compatible with osteomyelitis

**Fig. 10:**
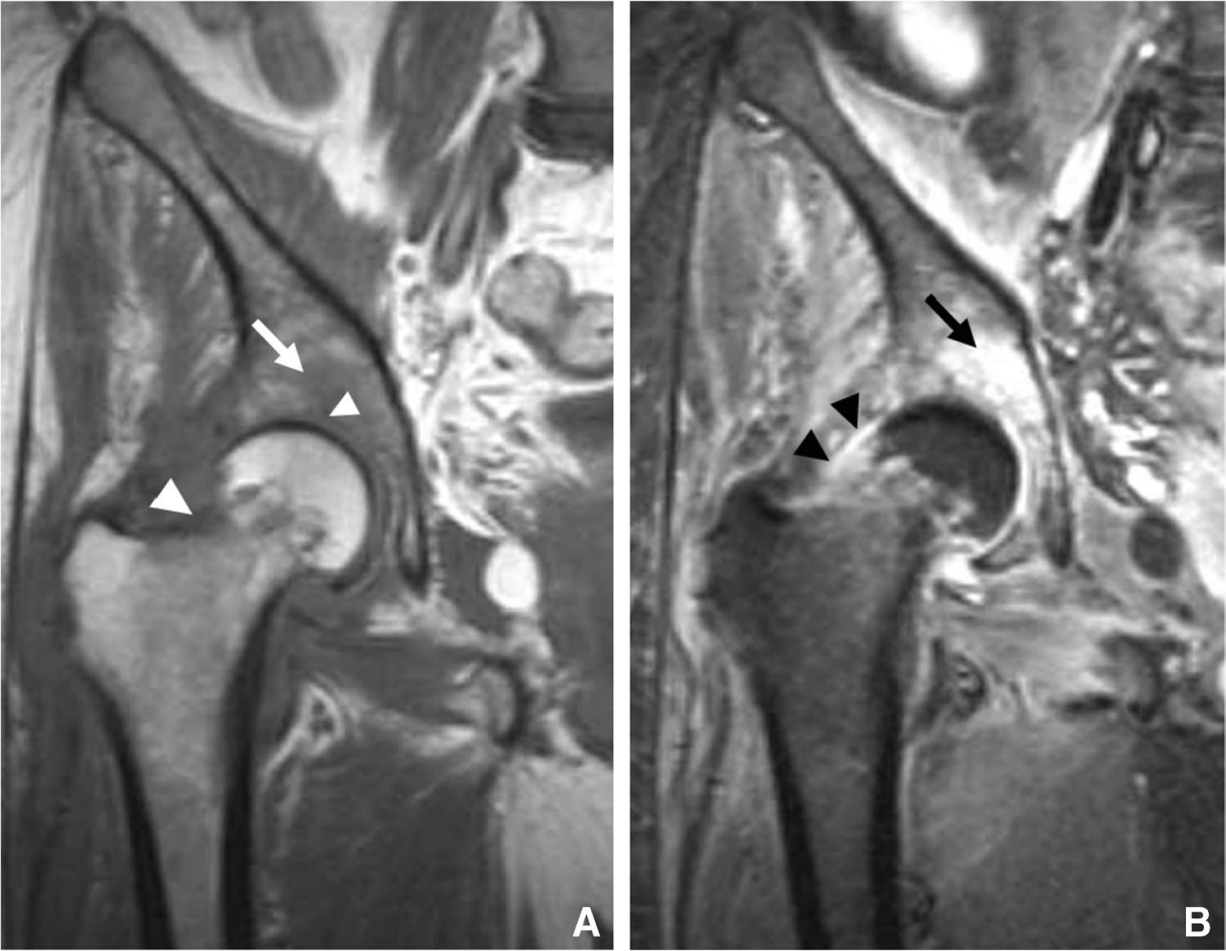
Septic arthritis with osteomyelitis in a 67-year-old male. Coronal T1 (**A**) and T1 fat-suppressed post-contrast (**B**) images of the right hip show erosions at the lateral femoral neck and superomedial acetabulum with disruption of the subchondral bone plate (arrowheads, **A**). Confluent replacement of normal fat signal in the medullary space of the adjacent acetabulum (arrow, **A**) with post-contrast enhancement (arrow, **B**) is consistent with progression to osteomyelitis. Enhancement of the joint fluid and capsule is compatible with synovitis (arrowheads, **B**)

Following contrast administration, due to synovial inflammation, the capsule and synovium show thick enhancement. Pericapsular edema and enhancement (edema and enhancement of the regional soft tissues subjacent to the joint capsule) help distinguish an infective etiology from chronic inflammatory arthropathies like rheumatoid arthritis (Fig. 9) [65]. A thin rim of subchondral edema-like signal in the underlying bone may be observed representing hyperemia. In later stages, erosions may occur at the margins of the joint, followed by frank bone destruction and osteomyelitis. Generally, when septic arthritis is associated with bone marrow edema-like signal extending into the medullary space, osteomyelitis should be suggested (Fig. 10) [65–72].

#### Controversy and rationale

Other inflammatory and crystal arthropathies, including rheumatoid arthritis, gout, psoriatic arthritis, and reactive arthritis routinely present with joint effusions, similar to septic arthritis. In addition, the source of the infection can be difficult or impossible to determine by imaging alone. Therefore, history and laboratory correlation are paramount. A monoarticular destructive arthropathy should be regarded as septic arthritis until ruled out, particularly in the setting of loss of joint space, poorly defined osseous margins, and a substantial joint effusion[62].

#### Recommendations

- Any monoarticular destructive arthropathy should be regarded as septic arthritis until ruled out. The terms *infectious arthritis* and *pyogenic arthritis* are rarely used.
- The term infectious or inflammatory arthritis may be appropriate when the diagnosis is uncertain.
- For the sacroiliac joints it is acceptable to combine the root term for infection with the joint name: the term *septic sacroiliitis* is part of common usage (use of *infectious sacroiliitis* is less common) [68, 71, 73]. Other specific terms for a particular infection of a particular joint have mostly become obsolete. To avoid confusion, we recommend that standard nomenclature for infection outlined in this article be applied uniformly when reporting MRI findings, with a differential regarding the infecting organism, if relevant.

### Synovitis

#### Definition and diagnosis

**Fig. 11:**
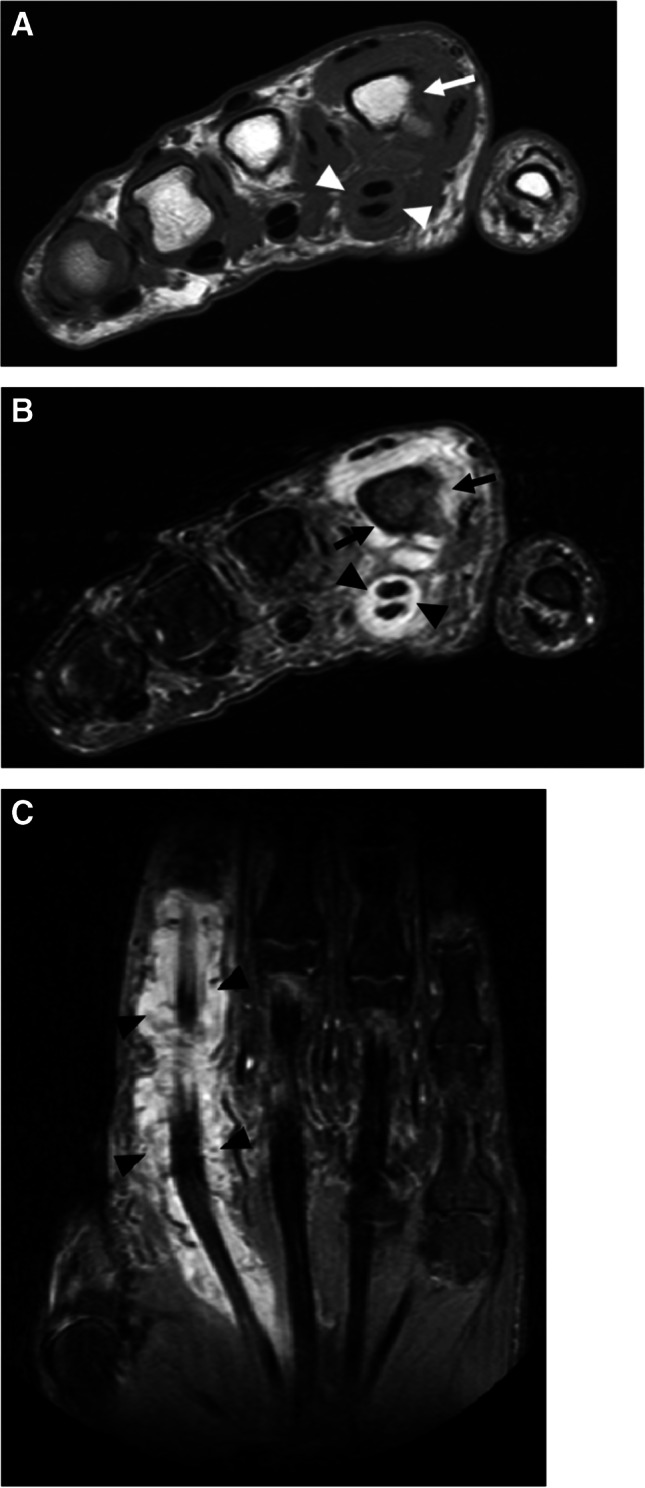
Septic tenosynovitis in a 48-year-old male. Axial T1 (**A**), T2 fat-suppressed (**B**), and coronal STIR (**C**) images of the hand show complex fluid distending the second digit flexor tendon sheath (arrowheads, **A**–**C**), compatible with septic tenosynovitis. Also present is a complex effusion of the second metacarpophalangeal joint (arrows, **B**), with erosion at the second metacarpal head (arrow, **A**), compatible with septic arthritis

Synovitis is inflammation of the synovial-lined spaces of joints, bursae, or tendon sheaths [49]. Normal synovium exists as a thin vascularized membrane, helping to create and maintain the joint or tendon sheath fluid through active transudation. Imperceptibly thin, normal synovium is barely visible on standard MRI. Inflamed synovium thickens and forms fronds extending into the joint fluid or tendon sheath, and is highly vascular, easily seen on MRI as a thick, irregular tissue at the inner margin of the joint capsule or tendon sheath, with thick post-contrast enhancement. In later stages, synovial fronds and synechiae extend into the joint fluid or tendon sheath (Fig. 11) [65, 69, 70, 72, 74].

#### Controversy and rationale

Synovitis is a nonspecific term that applies to infectious and noninfectious conditions. Care must be taken to communicate any concern for infection when applying the term in this context.

#### Recommendations

- Synovitis is in common usage for a variety of conditions; it is not specific for infection. Therefore, when it is used in an imaging report, we recommend it be accompanied by a differential diagnosis, including an estimation of risk of infection based on available information.

### Septic tenosynovitis/infectious tenosynovitis

#### Definition and diagnosis

Tenosynovitis is defined as inflammation of the tendon sheath [6], appearing as an abnormal tendon sheath fluid volume on MRI, while septic or infectious tenosynovitis implies infection of the tendon sheath. On MRI, septic tenosynovitis often demonstrates complex fluid signal with septations or synechiae (Fig. 11). Contrast-enhanced sequences demonstrate thick synovial enhancement and septations. Often there is ill-defined soft tissue edema-like signal around the sheath, reflecting hyperemia or capsular rupture and soft tissue spread. Abscesses or sinus tracts may be seen arising from the sheath. Septic tenosynovitis may be primary but more commonly arises from overlying ulceration or underlying septic arthritis [74–77].

A similar definition can be applied to other synovial based tissue such as bursae. Septic bursitis or infectious bursitis has been described with complex fluid signal in a distended bursa and thick rim enhancement following contrast administration [76, 78].

#### Controversy and rationale

Similar to septic arthritis, without history or laboratory findings the imaging appearance of septic tenosynovitis and septic bursitis overlaps the appearance of other non-infectious inflammatory conditions including rheumatoid arthritis, gout and psoriatic arthritis. Useful indicators supporting a diagnosis of septic bursitis or tenosynovitis include fever, overlying cellulitis, soft tissue swelling, and subjacent osseous erosion [62].

#### Recommendations

- Septic tenosynovitis and infectious tenosynovitis can be used interchangeably when MRI findings meet criteria in the setting of infection. For tendons with a paratenon instead of a tendon sheath (i.e., the Achilles), there is no equivalent term in common usage. In these cases, the term septic tenosynovitis is inappropriate; descriptive terms including infection of the involved tendon (i.e., infection of the Achilles tendon), or infectious paratenonitis are recommended.

### Erosion

#### Definition and diagnosis

Erosion is used to describe loss of subchondral bone plate integrity; for example, joint erosions in inflammatory arthopathies can be marginal, periarticular, or central depending on the etiology. Erosion implies a more active or rapid loss of the subchondral bone plate, as opposed to the term *scalloping*, or pressure erosion, which connotes slow remodeling of the bone resulting from juxtacortical mass effect, as can be seen with tenosynovial giant cell tumor. Erosion is also seen in the context of septic arthritis, initially at the bare areas at the joint margins, later progressing to more generalized articular surface destruction if left untreated. In prior work, erosions related to infection have been described as T2 marrow hyperintensity at the joint margins with variable T1 signal and disruption of the subchondral bone plate on all sequences (Figs. 9 and 10) [69, 72–74].

#### Controversy and rationale

As with *osteitis*, MRI findings described for *erosion* may actually represent the early stages of medullary involvement and osteomyelitis in the context of septic arthritis. Also, erosions are not specific for infection so without history or lab findings, a differential diagnosis of other crystal or inflammatory arthropathies would be considered. In addition, subchondral cyst-like lesions commonly observed in the setting of osteoarthritis may mimic an erosion, particularly if juxta-cortical in location, but should not have a discrete breach in the subchondral bone plate integrity.

#### Recommendations

- The term erosion in the context of septic arthritis can be used with the above findings, with the caveat that the finding could represent early osteomyelitis, especially if the T2 finding extends beyond the immediate subchondral bone.

## Bone surface

### Periosteal reaction, periostitis, and periosteal new bone formation

#### Definition and diagnosis

Involvement of the outer cortical layer, the periosteum, is a secondary sign of osteomyelitis [25, 62, 79–81]. Periosteal reaction, periostitis, and periosteal new bone formation are terms used interchangeably in the literature, seen in the setting of multiple underlying pathologic conditions, including both septic and sterile etiologies.

The periosteum is a layered membrane that protects underlying bone, supplies nutrients, regulated growth, and guides remodeling[49]. The periosteum histologically comprises two layers; an inner cambium layer, which is adherent to the bone surface, and an outer fibrous layer, which is adherent to the adjacent soft tissues [82]. The more vascular periosteum in younger patients is believed to partially account for differences in degree of periosteal reaction by age [62, 80, 81].

Periosteal reaction is defined as the reaction of periosteum to abnormal stimulants by forming new bone in distinctive patterns [49]. Periosteal reaction describes both processes extending in a centripetal fashion, such as direct spread of soft tissue infection, as well as those extending in a centrifugal fashion, including acute hematogenous osteomyelitis and bone tumors, the latter of which may only result in a lifting of the periosteum from the underlying cortex.

Periosteal reaction can be subdivided into aggressive and nonaggressive forms, with descriptive terms used to formulate a diagnosis on radiographs or CT. Specific appearances of the types of periosteal reaction can narrow the differential diagnosis of the underlying disease process in most pathologic entities [62, 79]. An accurate prospective diagnosis, however, may be occasionally difficult, particularly in the differentiation between Ewing’s sarcoma and osteomyelitis, which have overlapping imaging findings. One study found most imaging features on radiographs and MRI (including periosteal reaction) insufficient to serve as statistically significant predictors in making a diagnosis of either osteomyelitis or Ewing’s sarcoma [83].

In the setting of infection, periosteal reaction has been described as a common finding in both the acute and chronic setting and from both hematogenous and contiguous spread. It tends to me a more pronounced finding in osteomyelitis of childhood, in particular in the acute hematogenous form of the infancy period, due differences in local vascularity and degree of adherence to the underlying cortex over time [62, 79]. Various sources have described that the radiographic appearance of periosteal reaction may take anywhere between one to six weeks to develop [62, 79].

**Fig. 12:**
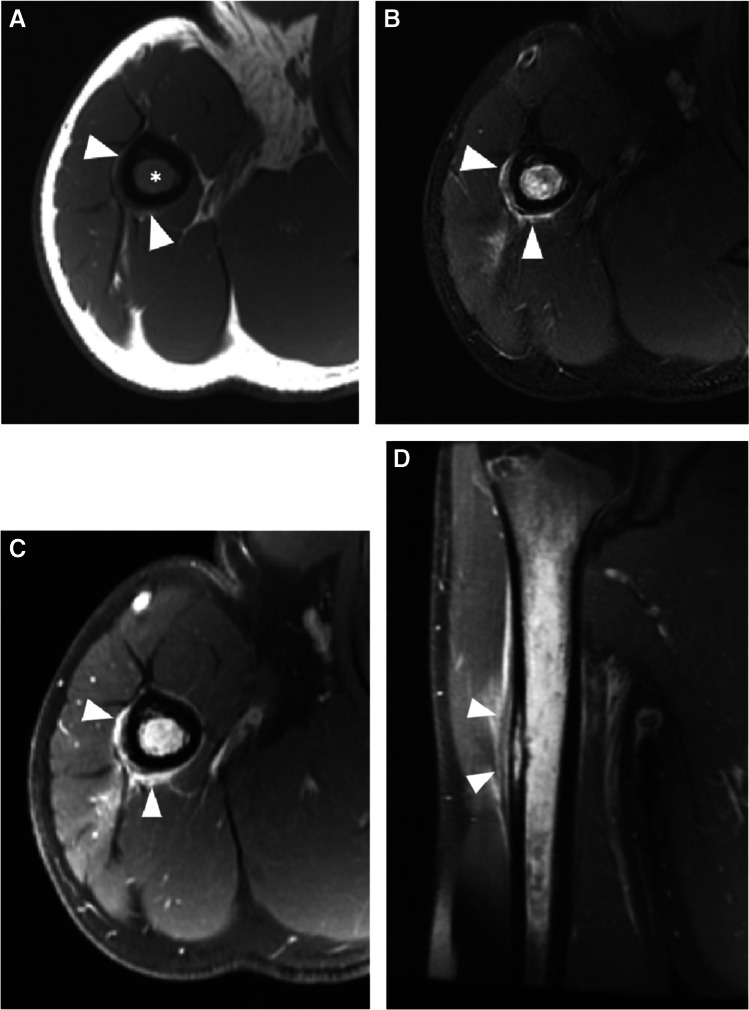
Humeral osteomyelitis with periosteal reaction in a 16-year-old male. Axial T1 (**A**), axial T2 fat-suppressed (**B**), and axial (**C**) and coronal (**D**) T1 fat-suppressed post-contrast images demonstrate confluent T1 marrow replacement of the humeral medullary canal (asterisk, **A**), compatible with osteomyelitis, with a thick rim of enhancing periosteal new bone formation (arrowheads), compatible with periosteal reaction

Primarily described as a radiographic and CT finding, periosteal reaction has rarely been described in the MRI literature. One such description is that of a low signal line separated from the underlying cortex by high signal fluid or pus (periosteal elevation) (Fig. 12) [25].

#### Controversy and rationale

Perhaps the paucity of discussion in the MRI literature reflects both the more subtle MRI appearance and the lack of a standardized definition of the finding on this lower resolution modality. Also, periosteal reaction becomes a more important finding on radiography and CT which have decreased sensitivity for making a diagnosis of osteomyelitis, when compared to the more specific bone marrow signal alterations which are easily identified on MRI, often obviating the need for description and detection of periosteal reaction on MRI.

#### Recommendations

- The terms periosteal reaction, periostitis, and periosteal new bone formation have previously all been used interchangeably and are all accurate descriptors of the underlying histopathologic process. For the purpose of standardization, the term periosteal reaction is recommended.

### Subperiosteal abscess

#### Definition and diagnosis

**Fig. 13:**
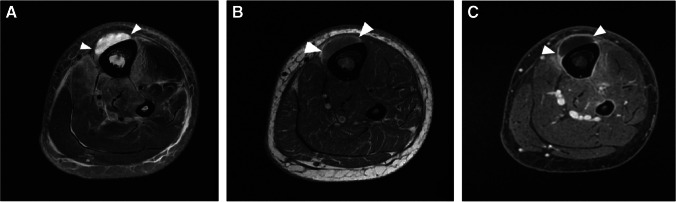
Subperiosteal spread of infection in a 21-year-old female with sickle cell disease and bone infarcts. Axial T2 fat-suppressed Dixon image with water amplification (**A**), axial T1 (**B**), and axial T1 fat-saturated post-contrast images of the lower leg demonstrating a subperiosteal fluid collection (arrowheads, **A**) which demonstrates a thin T1 hyperintense rim (“penumbra sign,” arrowheads, **B**) which enhances after contrast administration (arrowheads, **C**), confirming subperiosteal spread of infection with abscess formation

Subperiosteal abscess is an encapsulated fluid collection confined to the subperiosteal space. Subperiosteal abscesses are most commonly found in pediatric osteomyelitis, due to looser adherence of the periosteum to the underlying cortex [79], and is a significant prognostic finding often resulting in escalation to surgical management [84–87]. Subperiosteal abscesses have a higher association with pathologic fracture and higher morbidity, with postulation that the accumulated pressurized subperiosteal and medullary space septic material compresses the periosteal and endosteal vessels and results in necrosis [85] (Fig. 13). Subperiosteal abscesses are more frequently observed in children with Panton-Valentine leucocidin (PVL) gene positive staphylococcal infections [88]. Ultrasound has also been found to be a useful modality to establish the diagnosis and to follow patients with known subperiosteal abscess [86].

#### Controversy and rationale

It may be difficult to assess whether hyperintense material on fluid-sensitive images in the subperiosteal space represents an organized abscess, or a more primitive form of less defined infection (pre-abscess). For fluid collections confined to the subperiosteal space (not applicable to infection of the epidural space of the spine, which is beyond the scope of this article), this distinction does not appear to be clinically significant, as any form of spread of infectious material creates increased pressure and increased risk of necrosis and fracture, and both would intuitively be indications for surgical management.

#### Recommendations

- The term subperiosteal spread of infection is proposed as a more inclusive and accurate descriptor for all fluid collections confined to the subperiosteal space.

### Cloaca

#### Definition and diagnosis

In musculoskeletal infection, cloaca is defined as an opening or rupture of bony cortex overlying an area of osteomyelitis, allowing discharge of granulation tissue, intramedullary pus, or necrotic bone [89]. Cloaca may be seen in active or chronic infection and are often continuous with an intraosseous abscess. Intraosseous abscesses can decompress through defects that the infection creates in the cortex. Cortical breakthrough is a similar less specific overlapping term, defined as neoplastic and non-neoplastic bone destruction with transcortical extension, distinct from pathologic fracture.

**Fig. 14:**
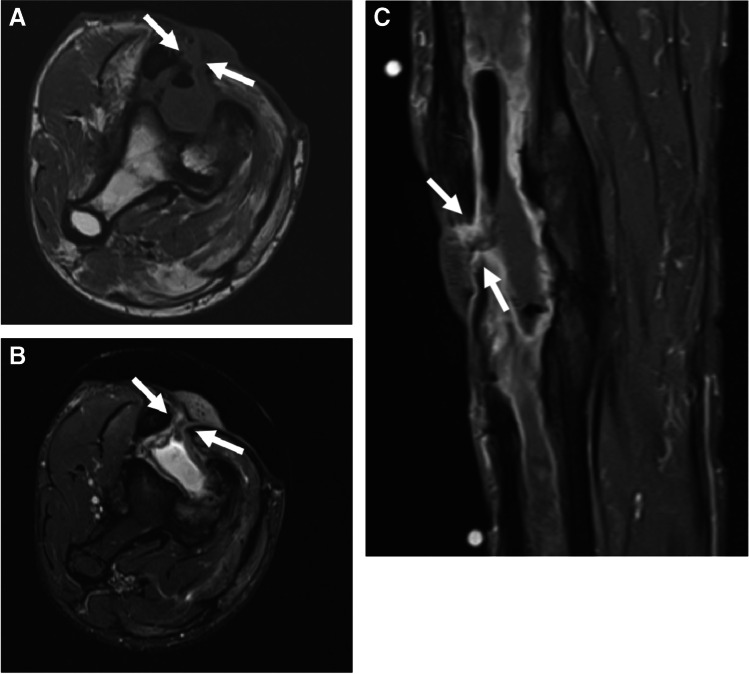
Cloaca and sinus tract in a 55-year-old male with chronic osteomyelitis. Axial T1 (**A**), axial T2 fat-suppressed (**B**), and sagittal T1 fat-suppressed post-contrast (**C**) images of the lower leg demonstrate chronic tibial osteomyelitis, with an intraosseous abscess decompressing to the skin surface via a cloaca and a contiguous sinus tract (arrows, **A**–**C**). Of note, the tibia and fibula are fused from prior trauma

On MRI, cloaca demonstrate hypointense signal on T1 weighted images and hyperintense signal on fluid-sensitive images. Cloaca may be limited to the cortex or may extend to the medullary cavity (Fig. 14).

#### Controversy and rationale

After an osseous infection is cleared, cloacal remnants often remain at the cortex. This is essentially reparative callus, but there can be a relative lucency at the site of the previous opening. Thus, it can be difficult to determine when a cloaca is closed on imaging.

Cloaca should be distinguished from pathologic fracture. While cloaca always result in discharge of infected soft tissue through a rent in the overlying cortex, the same is not always true of pathologic fracture. The term pathologic fracture should be used when there is a delineated fracture cleft resulting from weakened bone undergoing normal or minimal stresses. Cloaca should also be distinguished from an erosion, which should only be used to describe focal intra-articular defects of the subchondral bone plate.

#### Recommendations

- The term cloaca should be used an opening or rupture of bony cortex overlying an area of osteomyelitis.
- The nonspecific term cortical breakthrough is discouraged but may be used when the etiology is unclear. Cortical breakthrough should not be used to describe an infectious process.
- The term pathologic fracture should be used when there is a delineated fracture cleft resulting from weakened bone undergoing normal or minimal stresses.
- Consider MRI to identify intraosseous fluid collections (abscess) deep to the cloaca as in indicator of persistent infection.

## Medullary space

### Osteomyelitis

#### Definition and diagnosis

Osteomyelitis defines an infection of bone involving the medullary canal. Infectious osteitis is defined as a reactive change resulting from either an adjacent soft-tissue infection or a cortical (non-medullary) infection [25]. In contrast, the term osteitis, when applied to inflammatory arthropathies, implies inflammation of the medullary canal [90].

Foot osteomyelitis almost always occurs from contiguous spread of soft tissue infection-either from skin ulceration (i.e., in diabetic patients) or from a post-operative soft tissue defect [91]. The presence of ulcer can be an important secondary sign of osteomyelitis and improve diagnostic confidence [16, 28], and an ulcer with an area > 2 cm^2^ with a positive probe to bone test has positive predictive value for osteomyelitis [92–94].

Outside of the foot, osteomyelitis may be caused by hematogenous spread, spread from a contiguous infected source, direct implantation (i.e., penetrating injury or puncture), or after surgery [62]. An abundance of vascular channels with slow, turbulent flow within the medullary canal of the metaphysis or the metaphyseal equivalent predispose children to metaphyseal osteomyelitis through hematogenous spread. In infants, vessels which traverse the physis permit epiphyseal, and occasionally intraarticular spread of infection. Unlike infants and children, osteomyelitis of the tubular bones is infrequently observed in adults, who are more likely to develop osteomyelitis of the spine, pelvis, or small bones of the hands and feet. When osteomyelitis of a tubular bone occurs in the adult, the fused growth plate permits communication of metaphyseal and epiphyseal vessels and allows for subchondral spread of osteomyelitis, and occasionally complicating septic arthritis is observed [62].

Common organisms in children with hematogenous osteomyelitis include *Staphylococcus aureus* (both methicillin sensitive and methicillin-resistant isolates), *Kingella kingae*, *Streptococcus pyogenes*, and *Streptococcus pneumoniae* [88], while Gram-negative organisms may be observed in adults and intravenous drug users [62]. In the diabetic foot, *Staphylococcus aureus* is most commonly observed, followed by *Staphylococcus epidermidis* [4].

Radiographs are the preferred initial imaging modality for all patients with diabetic foot infection, with MRI recommended in patients requiring additional imaging, for example, if the diagnosis of osteomyelitis is uncertain based on radiographs, or to evaluate for concomitant soft tissue abscess [4]. MRI is the imaging modality with highest accuracy for detection of osteomyelitis, with prior meta-analysis demonstrating pooled sensitivity of 90%, and specificity ranging from 79 to 82.5% [95, 96].

**Fig. 15:**
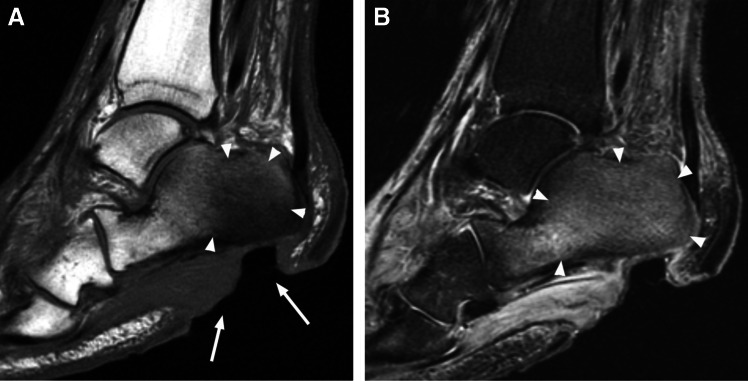
Osteomyelitis of the calcaneus in a 48-year-old diabetic female. Sagittal T1 (**A**) and STIR (**B**) images of the ankle show a large ulcer at the plantar aspect of the heel (arrows, **A**) communicating with the inferior calcaneus. Replacement of the normal calcaneal fatty marrow (arrowheads, **A**) and corresponding marrow edema-like signal (arrowheads, **B**) within the medullary space represents osteomyelitis

**Fig. 16:**
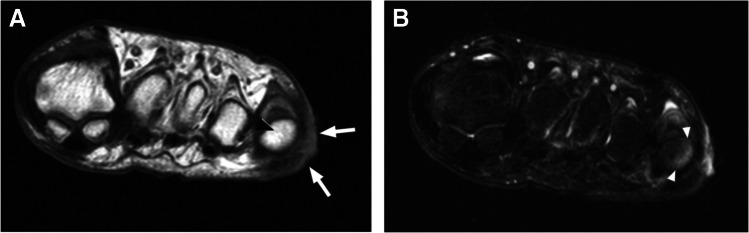
Marrow signal changes with high likelihood for osteomyelitis of the fifth metatarsal head in a 54-year-old diabetic female. Short axis T1 (**A**) and T2 fat-suppressed (**B**) images of the forefoot show lateral ulceration (arrows, **A**). Signal in the adjacent fifth metatarsal head is discordant- normal signal on T1 (arrowhead, **A**), with subcortical bone marrow edema-like signal on fluid sensitive images (arrowheads, **B**). In the presence of an adjacent soft tissue infection, findings should be considered to represent a high likelihood for early osteomyelitis

On MRI for suspected infection, osteomyelitis is diagnosed when marrow demonstrates low T1 signal (compared to the T1 signal of skeletal muscle), and high signal on fluid-sensitive images [97], with post-contrast enhancement (Fig. 15) [29]. Conversely, infectious osteitis has been described as demonstrating blurring or destruction of the low signal intensity cortex on all pulse sequences, with high T2 signal, and variable signal on T1 weighted images (Fig. 16).

**Fig. 17:**
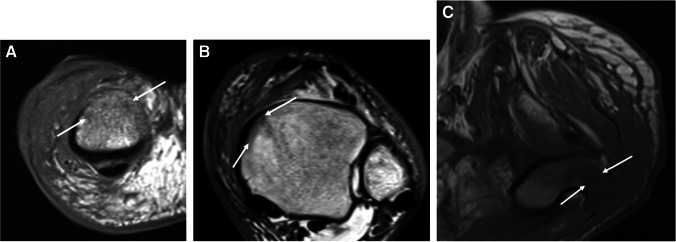
Patterns of T1 signal alteration. Short-axis T1-weighted image of the first proximal phalanx shows a hazy, reticular pattern of T1 marrow signal abnormality, where patchy areas of fat signal are seen amidst a background of reticular low T1 signal (arrows, **A**), while an axial T1 image of the ankle shows a subcortical distribution of signal abnormality, with a thin linear region of low T1 signal adjacent to the medial tibial cortex (arrows, **B**). An axial image of the hip shows T1 features compatible with osteomyelitis, with a confluent pattern and medullary distribution of marrow signal abnormality (arrows, **C**), involving a geographic area of the medullary canal along the greater trochanter

The appearance of T1 marrow replacement (low T1 signal relative to the signal of muscle) is crucial for high specificity for the diagnosis of osteomyelitis. Collins et al. found pedal T1 marrow replacement in a *confluent* pattern (contiguous and complete replacement of marrow signal) and a *medullary* distribution (low signal involving a geographic portion of the medullary canal), with concordant matching high signal on fluid-sensitive images in 100% of surgically proven cases of pedal osteomyelitis [98]. Conversely, in the same study, osteomyelitis was not observed in any patient with T1 marrow signal abnormality which was either subcortical in location (linear T1 marrow signal abnormality subjacent to the cortex, less than 3 mm thick) or in a hazy or reticular pattern (scattered foci of incomplete T1 marrow replacement) [98]. Howe et al. found similar findings of T1 marrow replacement in non-pedal osteomyelitis [99] (Fig. 17). Importantly, the subjects included in the studies by Collins et al. and Howe et al. all had tissue diagnosis from surgical biopsy or amputation and may represent more advanced cases of osteomyelitis.

It should be emphasized that the MRI appearance of osteomyelitis is dependent on both the imaging timepoint and regional soft tissue vascular integrity. Most pedal infections result from contiguous spread from an ulceration, involving first the subjacent soft tissues, the cortex, and finally the medullary canal. Subjects imaged prior to metabolization of fat within the medullary canal, either because of early onset of infection or because of insufficient or nonexistent tissue vascularity may fail to demonstrate the expected confluent, medullary T1 marrow replacement [20].

Conversely, marrow signal changes on fluid-sensitive images subjacent to an ulceration will appear earlier in the infection. The pattern and distribution of discordant marrow signal on fluid-sensitive images in this population has been only sparsely explored. In patients with discordant marrow signal, Sax et al. found a marrow/joint fluid ROI ratio of > 53% to be the strongest risk factor for developing osteomyelitis [100]. Collins et al. also found the intensity of signal on fluid-sensitive images relative to joint fluid to have predictive value, demonstrating marrow signal approaching that of joint fluid on fluid-sensitive images to have an 80% positive predictive value, relative to 38% positive predictive value for signal abnormality measuring less than joint fluid [98].

Osteomyelitis is considered acute when symptoms are present for < 2 weeks, and chronic when symptoms are present for > 4 weeks [97], with some studies describing an additional subacute phase with 1–3 months of symptoms [101, 102]. Aside from clinical history, MRI features are useful in predicting disease duration. Chronic osteomyelitis demonstrates inhomogeneous marrow signal, with areas of active disease demonstrating high signal on fluid-sensitive images, low T1 signal, interposed with areas of fibrosis which will demonstrate low signal on both T1 and fluid-sensitive images [102]. Brodie’s abscess (see section on “*Intraosseous abscess*”) is a feature specific for subacute or chronic osteomyelitis [36], while features of chronic osteomyelitis include cortical remodeling, sinus tracts, and sequestra [97, 102].

Intravenous contrast may be a useful triage tool to evaluate for osseous non-enhancement, whether it be from osteonecrosis, sequestrum, intraosseous abscess, or vascular insufficiency. Dry gangrene will appear as completely devitalized and often exposed bone, which will not show marrow edema or enhancement, yet is frequently infected. Importantly, intravenous antibiotics are unlikely to reach non-enhancing bone, and thus should be communicated to the referring clinician. The term infected, non-viable bone may be appropriate when there are findings diagnostic of osteomyelitis without contrast enhancement.

#### Controversy and rationale

Difficulty diagnosing osteomyelitis on MRI largely arises when marrow signal is discordant—high in signal on fluid-sensitive images without a matching confluent, medullary pattern of T1 marrow replacement. In suspected osteomyelitis, usage of the term osteitis or reactive marrow edema for discordant marrow signal, particularly when there are other imaging features with high positive predictive value for osteomyelitis (i.e., adjacent ulceration) is potentially misleading and may result in incorrect management. Furthermore, infectious osteitis has been classically described as infection limited to the cortex, although signal changes are observed in the medullary canal. Adding to confusion is the usage of the term osteitis in inflammatory conditions to describe bone inflammation involving the medullary canal.

T1-weighted images should be carefully scrutinized, as an accurate MRI diagnosis of osteomyelitis relies heavily on presence of confluent marrow signal abnormality in a medullary distribution. When T1 marrow signal is discordant, subcortical in location, or has a hazy, reticular pattern, secondary features should be actively sought after in order to determine and communicate likelihood of osteomyelitis to the referring clinician. Specifically, cutaneous ulcer and/or sinus tract adjacent to a marrow abnormality has a high positive predictive value for osteomyelitis [28]. This is supported by the findings of Duryea et al., who reported 61% of patients with discordant hyperintense signal on fluid-sensitive images either had an initial histologic diagnosis of osteomyelitis, or ultimately progressed to osteomyelitis [1].

Marrow signal may also be potentially contributed or caused by concomitant trauma, neoplasia, osteonecrosis, or arthropathies (including septic arthritis) or may be persistently abnormal after healing [97]. Erdmann et al. found marrow signal changes misinterpreted as osteomyelitis in 60% of uncomplicated septic joint infections [97].

Neuropathic osteoarthropathy frequently demonstrates marrow abnormality in the absence of infection and is another commonly encountered diagnostic dilemma [103]. Also, neuropathic osteoarthropathy and infection often coexist. In neuropathic arthropathy, presence of sinus tract, replacement of subcutaneous fat (indicating cellulitis), and joint erosion are secondary features associated with osteomyelitis, while thin rim enhancement of soft tissue fluid collections, presence of periarticular subchondral cysts, and intraarticular bodies support isolated neuropathic arthropathy without superimposed osteomyelitis [103]. Other features supportive of an isolated neuropathic arthropathy include multi-bone involvement with a periarticular distribution of marrow signal changes, bone fragmentation, signal abnormalities related to denervation within the regional musculature, and lack of ulceration. Again, if there is adjacent soft tissue infection and questionable marrow findings, it should be communicated to the clinical team that osteomyelitis is possible. On the other hand, in a diabetic patient with neuropathic disease, lack of adjacent soft tissue infection makes osteomyelitis unlikely.

Vascular insufficiency and tissue necrosis pose additional difficulty in the diabetic population. Vascularity is needed for fat metabolism required to produce confluent, medullary T1 marrow replacement, as well as measurable contrast enhancement. In a cohort of patients with non-enhancing (necrotic) tissue, Ledermann et al. found lack of T1 marrow replacement and marrow enhancement to be a source of false-negative imaging [20]. Morrison et al. also noted lack of vascular enhancement to be a source of false negative imaging in patients with chronic osteomyelitis [29].

In pediatric sickle cell patients, T1 marrow signal is not a reliable diagnostic indicator in differentiating between bone infarct and osteomyelitis [104]. Early osteomyelitis may also demonstrate normal T1 signal; fat metabolism and its disappearance in infection occurs more slowly than hyperemia, bone marrow edema, and cellular infiltration that results in hyperintensity on fluid-sensitive images and enhancement. Therefore, in the case of a discordant marrow pattern on MRI (T1 normal, bright on fluid-sensitive sequences) early onset of osteomyelitis may be proposed.

Clinical parameters should be reviewed to determine post-MRI likelihood of osteomyelitis. In difficult or uncertain cases, it is reasonable to recommend interval follow-up MRI.

#### Recommendations

- The term osteomyelitis is appropriate when concordant signal changes are present in the marrow on T1 and fluid-sensitive images.
- The term “high likelihood of osteomyelitis” should be used for any hyperintense marrow signal on fluid-sensitive images (regardless of T1 signal) adjacent to an ulcer, abscess, or sinus tract (or if there are other soft tissue features suggesting infection such as cellulitis).
- The term osteitis is nonspecific should not be used in the context of infection but should still be used in non-infection cases like those due to inflammatory arthritis.
- The term chronic osteomyelitis should be used if the marrow cavity demonstrates patchy areas of active disease and fibrosis, especially when coupled with features such as cortical remodeling, Brodie’s abscess, sequestrum, or sinus tract.
- The term infected, devitalized bone may be appropriate when there are findings diagnostic of osteomyelitis without contrast enhancement.

### Intraosseous abscess

#### Definition and diagnosis

**Fig. 18:**
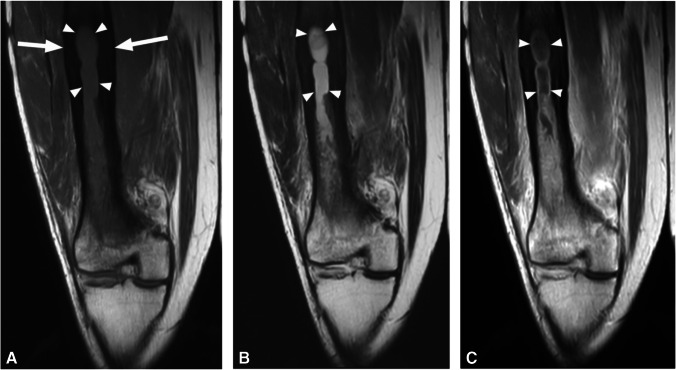
Intra-osseous abscess in a 35-year-old male with chronic osteomyelitis. Coronal T1 (A), T2 (B), and T1 post-contrast images (C) of the distal femur show cortical thickening (arrows, A) related to chronic osteomyelitis. A rounded region of low T1, high T2 signal (arrowheads, A, B) in the central medullary canal is present, revealing post-contrast rimenhancement (arrowheads, C); in the setting of infection, this meets criteria for intra-osseous abscess

An intraosseous abscess is an intraosseous pus-filled cavity with a rim of granulation tissue. Brodie’s abscesses are defined as circumscribed lesions found in subacute to chronic pyogenic osteomyelitis (most commonly of staphylococcal origin), having a predilection for (but not confined to) the ends of tubular bones [36, 62]. On MRI, an intraosseous abscesses cavity demonstrates high signal on fluid-sensitive sequences and low to intermediate T1 weighted signal, demonstrating peripheral enhancement after contrast administration (Fig. 18).

All cases should be scrutinized for the penumbra sign, a peripheral rim of hypervascular granulation tissue surrounding the abscess cavity, appearing as mildly T1 hyperintense signal about the low signal central abscess cavity, seen in 75% of intraosseous abscesses in a series by Grey et al. [101]. McGuiness et al. found the penumbra sign to have an average specificity of 96%, and a sensitivity of 27% for the identification of bone or soft tissue abscess, and along with high pre-test probability, this sign served as a useful feature in differentiating intraosseous abscess from neoplasm [35].

In cases of subacute to chronic osteomyelitis with Brodie’s abscesses, two discrete rings have been reported at the periphery of the abscess cavity. The inner ring is histologically composed of granulation tissue which is hyperintense on all series (penumbra sign) and demonstrates enhancement with contrast administration. The outer ring is hypointense on all series and is likely composed of eburnated bone and fibrotic reaction [36]. Grey et al. concluded that patients with this double line sign (as compared to an isolated single ring penumbra sign) had a more chronic stage of osteomyelitis [101].

The abscess occasionally traverses the physis and extends into the epiphysis in pediatric patients, although with effective antibiotic treatment, growth disturbance is rare [105].

DWI has been described as an adjunct diagnostic tool for the diagnosis of soft tissue abscesses, which demonstrate restricted diffusion, and may have a role, particularly in patients unable to receive intravenous contrast, although further studies are needed to determine utility for intraosseous abscesses [46].

#### Controversy and rationale

With clinical suspicion of osteomyelitis, the diagnosis of intraosseous abscess is usually straightforward. On rare occasions, difficulty arises in differentiating between abscess and neoplasia or other marrow replacing process. A cortically based abscess with perilesional sclerosis and periosteal new bone formation may mimic an osteoid osteoma. Presence of the penumbra sign and restricted diffusion are useful indicators supporting intraosseous abscess.

#### Recommendations

- The term intraosseous abscess is appropriate for intraosseous fluid-signal cavities with a rim of peripheral enhancement, or in the presence of restricted diffusion or the penumbra sign if contrast is not administered.
- The term Brodie abscess should be used for intraosseous abscesses in subacute or chronic osteomyelitis having a predilection for the ends of tubular bones.

## Necrosis

### Sequestrum

#### Definition and diagnosis

The presence of dead bone usually with fistulous tracts secondary to infection confirms the presence of chronic osteomyelitis [106]. The host’s inflammatory response including cytokines and leucocytes increase osteoclastic activity and lead to bone loss [107]. This devitalized bone becomes distinct from or “sequestered” from adjacent viable bone. Such nonviable, necrotic, and distinct bone contains bacteria which are protected from circulating antibiotics and is called a *sequestrum* [106, 108, 109]. Sequestra may be surrounded by granulation tissue [110]. More simplistically, a bony sequestrum is defined as a piece of devitalized bone that has become separated from the surrounding bone during the process of necrosis [111, 112]. Similarly, an area of osteonecrosis which becomes secondarily infected may be considered to be a functional sequestrum.

**Fig. 19:**
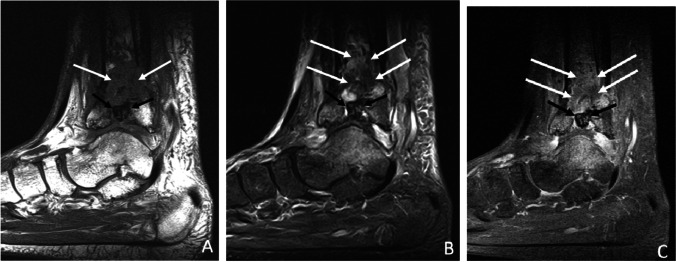
Sequestrum in a 40-year-old male with chronic osteomyelitis of the distal tibia following an open fracture. Sagittal T1 (**A**), STIR (**B**), and T1 fat-suppressed post-contrast (**C**) images of the ankle show destruction of the distal tibia with low T1 and intermediate-to-high signal STIR signal in the distal tibial medullary space, with heterogeneous enhancement (white arrows, **A**–**C**) representing chronic osteomyelitis. A focus of black signal (black arrows, **A**, **B**) at the articular surface represents a sequestrum, with no/minimal enhancement (black arrows, **C**) representing devitalization

While often seen on radiographs, sequestra are best identified with CT. A sequestrum appears as a focal area of mineralization surrounded by relative lucency on CT [109]. In cases where the diagnosis is uncertain, the addition of SPECT/CT or PET/CT may prove useful and will show a relative area of absent tracer accumulation corresponding to the sequestrum surrounded by increased tracer accumulation corresponding to the more viable infected tissue [113, 114]. The appearance of sequestra on MRI are not well described, but similar to infarcted, non-infected bone, sequestra would be expected to demonstrate similar marked low signal on T1-weighted images (Fig. 19). Importantly, the sequestrum, if identified as such, often in conjunction with CT imaging, should not enhance, especially if cortically based. However, peripheral enhancement due to granulation tissue surrounding the sequestrum may be possible [111].

#### Controversy and rationale

In a chronically infected patient, the presence of a sequestrum is definitive for chronic osteomyelitis. However, a sequestrum per se on an imaging study is not definitive of infection. Sequestra have been reported in primary lymphoma of bone, Langerhans cell histiocytosis, undifferentiated pleomorphic sarcoma, and occasionally metastatic disease. In addition, an osteoid osteoma or osteoblastoma may be confused for a sequestrum. Rarer lesions which have sequestra reported include chondroma and osseous lipomatous tumors [109, 111].

A clinical history of chronic infection is paramount to arriving to the diagnosis of bony sequestrum. If the diagnosis is unclear on MRI, CT should be recommended for further characterization.

#### Recommendations

- The term sequestrum should be used for an area of necrotic bone surrounded by viable, infected bone, often having a rim of granulation tissue.

### Involucrum

#### Definition and diagnosis

In the context of osteomyelitis, an involucrum describes the formation of a capsule of viable, new bone around an area of sequestered, necrotic bone. The involucrum can be viewed as a response to wall-off the necrotic, infected *sequestrum*. Depending on the location of the sequestrum, the involucrum may involve cancellous or cortical bone but often involves periosteal new bone formation.

The involucrum consists of different layers. The inner lining of the involucrum faces the sequestrum and consists of granulation tissue, which is often covered by a biofilm that protects bacteria from phagocytosis and humoral immunity [115]. The outer layer of the *involucrum* consists of expansile, coarse, woven bone, which is typically sclerotic in the mature stage. Eventually, the outer margin of the *involucrum* merges with the parental bone (Fig. 6).

The *cloaca* permits drainage of the sequestrum contents via a *sinus tract*, with its development indicating reactivation of infection. Eventually, sinus tracts perforate through the skin surface and decompress debris, bacteria, and pus. The size of an *involucrum* can increase substantially with persistent chronically active osteomyelitis.

In hematogenous osteomyelitis, the formation of an *involucrum* is more common in metaphyseal infections of infants and children, and less common in epiphyseal infections in adults.

The typical radiographic appearance of an *involucrum* is sclerotic, expansile bone that wraps around a sequestrum. The outer surface near periosteum may be coarse and irregular. The thickness varies depending on the length of time of the chronically active osteomyelitis, whereas increasing thickness over time suggests active infection [116]. A *cloaca* may present as focal partial-thickness defect (incomplete) or full-thickness perforation within the *involucrum* of varying sizes. On radiographs, the visibility of a cloaca depends on the location relative to the direction of the X-ray beam [117], whereas even small and incomplete cloaca are well visible on high-resolution CT images [118]. MRI can demonstrate and characterize a *cloaca* to better advantage, although the mineralized contents are less well visualized than on CT image. On MR images, the inner granulation tissue lining of the involucrum may demonstrate signal hyperintensity on T1-weighted MR images, like the penumbra sign described in subacute chronic osteomyelitis [119]. The signal intensities of the osseous component of the *involucrum* vary and include edema pattern on fluid-sensitive images, and hypo- or hyperintensity on T1-weighted MR images depending on the amounts of marrow fat contents and sclerotic bone [120].

Curative surgical intervention is usually referred to as *debridement*, which refers to the removal of infected and necrotic bone and tissues. During surgery, *sequestrectomy* and the complete opening of the *cloaca* are essential to incite healing. Thus, cases with potential involucrum should be carefully inspected for the presence of sequestrum and cloaca, which should be communicated to the ordering team, with CT and MRI playing potential complementary roles in optimizing diagnostic accuracy. In contrast, resection of the *involucrum* is not required but can be performed to correct deformities and to avoid the formation of a new sequestrum within the remaining *involucrum*. Local treatment of the resulting bone cavity includes antibiotic beads and cancellous bone grafts.

#### Recommendations

- The term involucrum should be used to describe a capsule of viable, new bone which forms around an area of necrotic (sequestered) bone.

## Conclusion

This consensus statement and suggested nomenclature reflect the underlying pathophysiology and clinical relevance of MR imaging findings of musculoskeletal infection outside of the spine more precisely than terms in current use, aiming to improve effective communication across clinical specialties in order to improve patient care.
