# Correction to: MRI nomenclature for musculoskeletal infection

**DOI:** 10.1007/s00256-022-03999-6

**Published:** 2022-01-27

**Authors:** Erin F. Alaia, Avneesh Chhabra, Claus S. Simpfendorfer, Micah Cohen, Douglas N. Mintz, Josephina A. Vossen, Adam C. Zoga, Jan Fritz, Charles E. Spritzer, David G. Armstrong, William B. Morrison

**Affiliations:** 1 NYU Langone Medical Center, New York, NY USA; 2 UT Southwestern Medical Center, Dallas, TX USA; 3 Cleveland Clinic, Cleveland, OH USA; 4 Albert Einstein Medical Center, Philadelphia, PA USA; 5 HSS, New York, NY USA; 6 VCU, Richmond, VA USA; 7 Thomas Jefferson University Hospital, Philadelphia, PA USA; 8 Duke University Hospital, Durham, NC USA; 9 Keck Hospital of USC, Los Angeles, CA USA

**Correction to: Skeletal Radiology (2021) 50:2319–2347**

https://doi.org/10.1007/s00256-021-03807-7

The article “MRI nomenclature for musculoskeletal infection”, written by Alaia, E.F., Chhabra, A., Simpfendorfer, C.S., Cohen, M., Mintz, D.N., Vossen, J.A., Zoga, A.C., Fritz, J., Spritzer, C.E., Armstrong, D.G., Morrison, W.B., was originally published Online First without Open Access. After publication in volume 50, issue 12, page 2319–2347 the author decided to opt for Open Choice and to make the article an Open Access publication. Therefore, the copyright of the article has been changed to © The Author(s) 2022 and the article is forthwith distributed under the terms of the Creative Commons Attribution 4.0 International License, which permits use, sharing, adaptation, distribution and reproduction in any medium or format, as long as you give appropriate credit to the original author(s) and the source, provide a link to the Creative Commons licence, and indicate if changes were made. The images or other third party material in this article are included in the article’s Creative Commons licence, unless indicated otherwise in a credit line to the material. If material is not included in the article’s Creative Commons licence and your intended use is not permitted by statutory regulation or exceeds the permitted use, you will need to obtain permission directly from the copyright holder. To view a copy of this licence, visit http://creativecommons.org/licenses/by/4.0. Open access funding enabled and organized by Projekt DEAL.

The original articles has been corrected.

